# Developmental trajectory of communication repair in children with Fragile X Syndrome

**DOI:** 10.1177/2396941520909014

**Published:** 2020-02-27

**Authors:** Heather Fielding-Gebhardt, Steven F Warren, Nancy C Brady

**Affiliations:** Child Language Doctoral Program, University of Kansas, Lawrence, KS, USA; Department of Speech-Language-Hearing: Sciences and Disorders, University of Kansas, Lawrence, KS, USA; Department of Speech-Language-Hearing: Sciences and Disorders, University of Kansas, Lawrence, KS, USA

**Keywords:** Pragmatics, Fragile X, language development, social communication

## Abstract

**Background and aims:**

The development of communicative competence requires both language and social skills. The ability to repair following a communication breakdown is critical for continued conversational interchange and to ensure comprehension of bids for communication. Communication repair demonstrates adequate language and social skills. Children with Fragile X Syndrome have difficulty with language development and social skills, which may result in delays or deficits in repair. Repair may be additionally impaired in children with Fragile X Syndrome and co-morbid autism. This study examined the development of repair in children with Fragile X Syndrome from toddlerhood into middle childhood.

**Methods:**

Fifty-five children with Fragile X Syndrome and their biological mothers participated. Data were collected during in-home visits approximately every 18 months. Videotaped mother–child interactions were collected, as well as standardized assessments of language, social skills, and autism symptomology.

**Results:**

Children with Fragile X Syndrome acquired the ability to repair at 90% mastery by three-and-a-half years of age. Multilevel logistic regressions predicting probability of repair indicated marginally significant effects of mean length of utterance and number of different words, and significant effects of global social skills and autism symptomology. Effect sizes were small to moderate.

**Conclusions:**

Ability to repair was measured in a naturalistic setting, which allowed children with Fragile X Syndrome to utilize repairs in their daily interactions. Although children with Fragile X Syndrome may have delayed development of repair relative to typically developing expectations, in general they nonetheless catch up and demonstrate a robust ability to repair by three-and-a-half years of age. However, this study provides evidence that individual differences in language and social skills may influence ability to repair in children with Fragile X Syndrome. Finally, the relationship between autism symptoms and repair remains unclear, necessitating further exploration.

**Implications**: Given the noted delay in repair in young children with Fragile X Syndrome, clinicians working with this population should target development of this skill as early as possible to maximize successful social interactions. This may be particularly necessary for children with Fragile X Syndrome and co-morbid autism.

## Introduction

Clarity is essential for successful communication between two speakers. Grice (1975) proposed four maxims to guide conversation, the last of which is the maxim of Manner. If a speaker violates this maxim, they risk unclear communicative signals leading to communication breakdowns and the need for communication repair. Once a communication breakdown has occurred and the listener signals their noncomprehension of the speaker’s utterance, the speaker is then obliged to repair their original utterance. Repair is a relatively complex pragmatic skill in which the speaker must monitor their communication partner and be prepared to modify their original message. If the speaker struggles with social communication or expressive language, s/he may have difficulty repairing. The current study examined the development of repair in children with Fragile X Syndrome (FXS), a genetic neurodevelopmental disorder associated with delayed language development and impaired pragmatic and social communication skills (Abbeduto et al., 2007; Finestack et al., 2009; Klusek et al., 2014; Lewis et al., 2006; Mazzocco et al., 2006; McDuffie et al., 2012; Roberts et al., 2007; Sterling & Abbeduto, 2012).

Fundamentally, repair is a social communication skill, and the ability to repair requires adequate linguistic and social skills. Wetherby and Prizant (2003) define repair as the “ability to persist in communication and to modify a signal when a goal is not obtained” (p. 38). The speaker must evaluate their conversational partner’s comprehension during discourse and adjust their own communication accordingly. This requires constantly monitoring one another’s signals, shifting perspectives between self and other. The ability to repair following a communication breakdown is critical to continued conversational interchange and demonstrates one’s communicative competence (Alexander et al., 1997). Repairing requires syntactic, semantic, phonological, and lexical knowledge, as well as Theory of Mind, social awareness, nonverbal communication, emotion regulation, and persistence (Alexander et al., 1997).

Repair unites the need for language development and social communication. The development of intentional communication in children, along with increasingly diverse lexicons and complex morphosyntax, forms the building blocks for conversation. Typically, this takes the form of verbal speech, supplemented by nonverbal communication strategies such as socially modulated eye contact, joint attention, and gesture use. The ability to repair relies on the development of intentional communication and the integration of speech, gestures, and nonverbal communication. The social use of language, also termed pragmatics, enables us to successfully participate in conversations, as we use language for a variety of reasons (greeting, demanding, etc.), in different contexts (setting, conversational partner, etc.), and to follow the rules of conversation in our culture (turn-taking, topic-maintenance, repairing, nonverbal communication; ASHA, 2019). The social ability to shift perspectives and monitor a conversational partner’s signals is critical for repair. As children mature, they develop Theory of Mind, or the ability to recognize that others have their own unique desires and experiences, and that others behave in accordance with their personal desires and experiences. Theory of Mind is linked to repair because once it begins developing, young children begin to recognize noncomprehension signals and the need for repair (Alexander et al., 1997).

Typically developing (TD) children acquire repair strategies in coordination with the acquisition of intentional communication and demonstrate increasing complexity of repair strategies with maturation and as language develops (Alexander et al., 1997; Golinkoff, 1986). As children begin to recognize the social utility of intentional communication, they may also begin to understand the social cues that indicate they should repair. The ability to repair is acquired relatively early and is a robust skill. In fact, children in the one-word stage (14.8 months old on average) and children in the multi-word stage (21 months old on average) repair 90% of the time when required (Alexander, 1994; Alexander et al., 1997; Gallagher, 1977).

However, in a study of young children with severely delayed expressive language and below average IQ scores, Brady et al. (2005) found that the percentage of repair ranged from 33% to 70% (mean = 58%). The children in their higher language group, who functionally used between 6 and 12 words, symbols, or signs, repaired more often than those in the lower language groups (Brady et al., 2005), suggesting an effect of expressive language. Yet, this may not be true in all populations. Adults with limited language skills are relatively strong at repairing (Brady et al., 1995). Brady et al. (1995) found that in a sample of 28 adults with severe or profound intellectual disability who communicated primarily through gestures and vocalizations, 25 repaired at least once following a breakdown. Specifically, they repaired following 74.52% of breakdowns in protoimperative initiations, and 55.6% of breakdowns following protodeclaratives (Brady et al., 1995). Although these adults had very limited communication skills, they were still able to repair when necessary, albeit at a lower rate than expected. Thus, the association between expressive language and repair remains unclear.

When children have impairments in social communication, such as delayed onset of speech or intentional communication, delayed syntactic development, limited vocabulary, and difficulty adhering to social rules of language use, they may also have difficulty with repair. Children with FXS demonstrate delayed language across receptive, expressive, and pragmatic domains that varies widely and across gender (Abbeduto et al., 2007; Finestack et al., 2009; Klusek et al., 2014; Lewis et al., 2006; Mazzocco et al., 2006; McDuffie et al., 2012; Roberts et al., 2007; Sterling & Abbeduto, 2012). Studies of communication breakdowns in children with FXS have found impairments in ability to signal noncomprehension during adolescence (Abbeduto et al., 2008; Martin et al., 2017). Abbeduto et al. (2008) found that adolescent males and females with FXS signal communication breakdowns significantly less than mental-age-matched same-sex TD peers during a contrived task. Building on this, Martin et al. (2017) examined noncomprehension signaling in children and adolescents with FXS and autism. Children and adolescents with FXS and autism signaled communication breakdowns less often than those with FXS-only, suggesting an effect of autism symptomology on repair.

Although Roberts et al. (2002) reported that boys with FXS-only between 33 and 65 months old demonstrated a weakness in repairing, relatively little is known about repair in children with FXS. One study suggested that children and adolescents with FXS and FXS with co-morbid autism were strong repairers (Barstein et al., 2018). However, this study’s participants varied greatly in age, such that strong repair skills may have been driven by older participants. Beyond these two studies, the ability to repair, and the development of this ability, has not been studied in FXS. Accordingly, the development of repair in young children with FXS needs further investigation. Additionally, given the potential effect of autism symptomology on signaling noncomprehension, further research is needed to determine the effect of autism symptomology on repair.

Repair is a social communication ability that demonstrates skillful use of language and social skills. With limited ability to repair, individuals may experience communicative difficulties in a variety of settings. This becomes especially true when individuals with speech or language difficulties struggle to repair. To our knowledge, there have been no studies examining the developmental trajectories of repair in children with FXS. Therefore, our purpose was to examine the development of repair in young children with FXS and to explore the association between expressive language, social skills, autism symptomology, and repair. Thus, we asked two questions:

1. What is the developmental trajectory of repair in children with FXS, and is it delayed?
2. How does variability in expressive language, social skills, and autism symptomology account for differences in repair within and across children with FXS?

Given that repair likely develops in tandem with intentional communication and social skills, we predicted that the development of repair would be associated with the development of expressive lexical diversity (vocabulary), expressive syntactic complexity, and social skills. Additionally, we predicted that higher autism symptomology would be associated with impaired repair based on past findings that demonstrated associations between autism symptoms and repair or noncomprehension signaling in children with FXS and co-morbid autism (Abbeduto et al., 2008; Barstein et al., 2018; Martin et al., 2017).

## Method

### Participants

Fifty-five children with FXS (11 females and 44 males) and their biological mothers were studied from toddlerhood into late childhood (Brady et al., 2014; Warren et al., 2010). Measures were collected from each dyad at five time points. At Time 1, children averaged 28.60 months of age (±9.21) and at Time 5, children averaged 109.45 months of age (±9.26). Ages at each time are reported in Table 1. Families were recruited through advertising at national conventions and via an FXS parent list server, as well as networking with FXS family support groups. Participants were also recruited through a national research registry housed at the University of North Carolina-Chapel Hill. Because FXS is a rare disorder, this sample is one of convenience. However, there was moderate diversity on socioeconomic status, maternal education, and maternal marital status (we refer the reader to previous publications from this dataset, listed above).

**Table 1.** Descriptive statistics for predictors.

Variable	Time	Mean (SD)	Range
Age (months)	1	28.60 (9.21)	11–48
	2	49.11 (8.91)	26–64
	3	61.11 (8.83)	40–76
	4	91.64 (7.99)	75–104
	5	109.45 (9.26)	88–138
Number of different words	1	19.82 (30.09)	0–119
	2	56.47 (46.42)	0–159
	3	74.52 (36.99)	0–189
	4	158.21 (78.14)	0–304
	5	166.38 (82.38)	1–318
VABS social domain raw score	1	34.90 (8.25)	23–61
	2	45.73 (8.82)	28–69
	3	52.93 (10.77)	31–79
	4	65.71 (17.02)	31–108
	5	70.17 (18.96)	24–103
CARS score	1	25.60 (5.89)	15.5–42
	2	26.57 (5.67)	16.5–36.5
	3	26.56 (5.98)	16–39
	4	25.78 (6.86)	16–44.5
	5	26.05 (6.25)	15.5–42.5
Mean length of utterance (morphemes)	1	0.82 (.60)	0–1.93
	2	1.49 (.54)	0–2.75
	3	2.29 (.83)	0–5.17
	4	1.99 (.86)	0.11–3.63
	5	2.01 (.88)	0.11–3.72

SD: standard deviation; VABS: Vineland Adaptive Behavior Scales; CARS: Childhood Autism Rating Scale.

### Procedure

Each dyad was visited in their home roughly every 18 months. During the data collection visits, trained examiners (typically graduate students) administered a battery of standardized assessments. Following this, the children and their mothers participated in several structured interactions that were videotaped. Each of the following contexts was videotaped for 5 min: reading a book together, making and eating a snack together, and either free play or making a craft together. Together these contexts resulted in a child communication sample that was 15 min long, which is an adequate length based on methodologies in previous studies of communication development in children with FXS or other neurodevelopmental disorders (Brady et al., 2014; Warren et al., 2010; Yoder et al., 2015).

### Measures

The mother–child interactions were digitized and then coded using Noldus™ Observer software (Noldus Technology, 2002). We coded both the mother and child behavior-by-behavior. From the child coding, we obtained syntactic complexity, lexical diversity, and probability of repair. Syntactic complexity was measured as mean length of utterance (MLU) in morphemes, and lexical diversity was number of different words (NDW) produced by the child. Repair was defined as the child clarifying the intended message in the form of a mother-directed verbalization (word), vocalization, gesture, and/or sign following a maternal communication breakdown. Repair could be delivered in different modalities or in a combination of modalities. For example, a child may repair using a gesture alone, or by pairing a gesture with a vocalization. Probability of repair was defined as the number of times the child repaired when faced with the opportunity to do so (when there was a communication breakdown) and was calculated as total number of repairs/total number of mom communication breakdowns.

From the mother coding, we obtained the number of communication breakdowns signaled by the mother and maternal responsivity, which was a composite of her comments, requests for verbal complies, and child-directed praise. Communication breakdowns occurred when the mother signaled noncomprehension of the child’s turn (vocalization, verbalization, sign, or gesture). Signaling noncomprehension could be in the form of a request for verbal compliance (“what did you say?), a marker of noncomprehension (“huh?”, “hmm?”), or an ignore.

Graduate students and researchers were trained to identify and code communication breakdowns and repairs to a training criterion of 80% agreement across contexts. Once they reached this training criterion, they independently coded mother and child behaviors. Mother and child coders compared transcripts and resolved disagreements through consensus. For more information on reliability procedures, we refer the reader to earlier publications (Brady et al., 2014; Warren et al., 2010).

#### Social skills

The Vineland Adaptive Behavior Scales (VABS; Sparrow et al., 1984; Sparrow et al., 2005) is a standardized, semi-structured parent interview assessing communicative, social, daily-living, and motor skill functioning in children and adults. Scores from these four domains are summed to create the Adaptive Behavior Composite, which is a score of an individual’s overall adaptive behavior. The interview takes between 20 and 60 min to administer. Each item is scored along a three-point Likert scale indicating if the child never, sometimes/partially, or usually performs a behavior. Mothers completed this assessment about their child with a trained interviewer during each home visit. Raw scores from the Socialization domain at each time were included in this analysis as measures of social skills. Raw scores were preferred over standard scores because they are sensitive to growth and change over time, whereas standardized scores are not.

#### Autism symptomology

The Childhood Autism Rating Scale (CARS; Schopler et al., 1988) was used to assess autism symptomology. This assessment measures autistic behaviors on a four-point Likert scale, and it was completed by the examiners following each home visit. Higher scores indicate more severe symptoms of autism, with scores over 30 suggesting mild-to-moderate symptomology. Scores from each time were included in the analysis.

## Results

### Analysis

The extent to which children with FXS repaired following maternal communication breakdowns was examined in a series of multilevel models. We used full-information maximum likelihood estimation based on LaPlace approximation to conduct multilevel binomial models run in SAS software (SAS Institute Inc., 2013) version 9.4 with PROC GLIMMIX. Multilevel modeling is a form of regression analysis that allows us to examine change over time while considering the impact of within- and between-person predictors. Multilevel modeling also allows us to consider the influence of time-varying and time-invariant predictors. Each predictor was apportioned into between-person and within-person effects, also termed Level-2 and Level-1 effects, respectively, using person mean centering (Hoffman, 2015). In this type of centering, the Level-2 predictor represents the person’s average score across occasions (Times 1–5), and the Level-1 predictor represents the difference between the person’s average score and their score at each occasion. Thus, for each predictor, the Level-2 effect demonstrates how a person performs on average and the Level-1 effect demonstrates how far that person deviates from their own mean at each occasion. Finally, we centered age at 2 years, so that intercepts were meaningful and indicated expected probability of repair at 2 years.

We predicted the number of times the child repaired relative to the number of opportunities she/he had to do so, and we refer to this variable as probability of repair. Because this results in a non-normal outcome distribution, we used a binomial conditional outcome distribution with a logit link function to constrain the predicted proportion between 0 and 1. The models thus predict the logit (log-odds) of a successful repair for a given trial which can be translated into a proportion correct via an inverse link function. Fixed effects can be interpreted as unit-specific, and the significance of fixed effects was evaluated with Wald tests (*t*- or *F*- tests using between-within denominator degrees of freedom). The significance of random effects was evaluated via likelihood ratio tests. However, the small sample size limited our ability to include multiple random effects beyond a random intercept and a random effect accounting for overdispersion.

All dyads contributed data from each time point. However, we excluded data from occasions during which there were fewer than two mother-signaled communication breakdowns. Only occasions in which the child had multiple opportunities to repair were included in the analyses because we wanted to avoid spurious effects due to limited opportunities to respond. Thus, sample size at each occasion reflects the number of dyads with two or more opportunities to repair at that occasion. Had we chosen to keep all occasions, a high proportion of data points with only one communication breakdown and thus only one opportunity to respond may have disproportionately positively skewed our data. Indeed, in our preliminary analyses, we saw that inclusion of single communication breakdowns in the dataset skewed our data such that participants seemed more responsive to requests for repair.

### Descriptive statistics and correlations

Descriptive statistics for each predictor are provided in Table 1. NDW and VABS social scores increased over time. MLU also increased, but there was a slight decrease following Time 3. Average CARS scores were stable over time. Relatively high standard deviations and ranges suggest considerable variability between children. Table 2 shows correlations between variables collapsed over time. Probability of repair was significantly positively correlated with age, MLU, and VABS social skills, although these correlations were small. Probability of repair was not correlated with NDW or CARS scores. Although not related to our research questions, it is interesting to note the strong, significant correlations among the predictors, suggesting age-related increases in language and social skills, and negative associations between autism symptoms and language and social skills.

**Table 2.** Correlations.

	Age	MLU	NDW	VABS social	CARS
Repair	.15*	.20**	.10	.21**	.05
Age		.21**	.58**	.61**	.07
MLU			.43**	.37**	−.22**
NDW				.70**	−.41**
VABS social					−.43**

MLU: mean length of utterance; NDW: number of different words; VABS: Vineland Adaptive Behavior Scales; CARS: Childhood Autism Rating Scale.

*Correlation is significant at the 0.05 level (2-tailed).

**Correlation is significant at the 0.01 level (2-tailed).

To investigate potential differences in repair based on co-morbid autism, we conducted a *t*-test to examine the difference in means for children with FXS-only and children with FXS who had CARS scores greater than 30 (which suggests co-morbid autism). The effect of autism diagnosis on probability of repair was not significant (*t *=* *0.69, *p *=* *0.49).

### Developmental trajectories of repair

**Figure 1.**
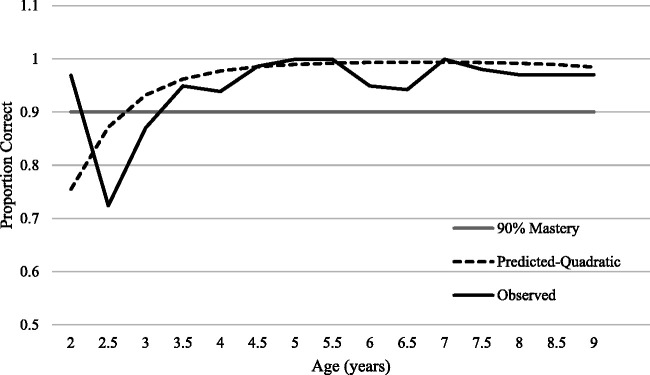
Model predicted and observed growth trajectory for mean probability of repair.

Relative to an empty model, our first step in modeling suggested a need for both a random intercept variance for participants, −2ΔLL(1) = 1997.1, *p *=* *0.00, and an additive offset to the Level-1 binomial-predicted variance, −2ΔLL(1) = 3341.0, *p *=* *0.00. The Level-2 random intercept suggests differences in probability of repair between children at 2 years and the need to account for differences between children. Visual inspection of growth trajectory of probability of repair suggested a quadratic effect of time, see the observed growth trajectory in Figure 1. So, we added both a linear and quadratic effect of time to the model. The addition of fixed linear and quadratic effects of age in years suggested significant growth in repair over time, *F*(1,133) = 10.55, *p *<* *0.01, and *F*(1,133) = 8.47, *p *<* *0.01, respectively. The effects of linear and quadratic time suggest that probability of repair increases with age, but that the rate of increase slows with age. This is consistent with a plateau of ability, which is expected given that probability of repair cannot exceed 1. Effect size, calculated as an odds ratio (Chen et al., 2010; Ialongo, 2016), suggests that for every 3 months older, the probability of repair increases 1.09 fold.

Model predicted and observed mean percent correct repair over time are shown in Figure 1. Observed probability of repair at 2 years was 0.96, and model predicted was 0.72. However, there was a decrease in observed probability of repair at 2.5 years (to 0.72) followed by a quadratic increase. Thus, we believe that the high probability of repair at 2 years was due to a combination of high variation and a small sample at that early age and does not accurately represent ability early on. The inclusion of an additive offset to the Level-1 predicted variance assists in accounting for the high variability at that age and was retained in all subsequent models. Both model predicted and observed probability of repair was over 0.9 by 3.5 years, suggesting that children with FXS robustly demonstrate and maintain the ability to repair by 3.5 years.

### Language and social factors affecting development of repair

The addition of expressive language effects (Level-2 and Level-1) suggested that between-person average MLU and NDW were marginally significant predictors of probability of repair, see Table 3. Specifically, Level-2 MLU was nearing significance, *F*(1,51) = 3.43, *p *=* *0.07, as was Level-2 NDW, *F*(1,51) = 2.94, *p *=* *0.09. Similarly, within-person fluctuations in MLU were marginally significantly predictive of probability of repair, *F*(1,130) = 3.05, *p *=* *0.08. Within-person fluctuations in NDW were not significantly predictive of probability of repair, *F*(1,130) = 1.58, *p *=* *0.21. Although the effect of Level-2 MLU was marginally significant, odds ratio estimates suggest that a one unit increase in average MLU (e.g., from 0 to 1.0, or 1.5 to 2.5) yields a probability of repair that is 6.62 times greater. Thus, children who had higher MLUs on average had a much higher probability of repairing following a mother-signaled communication breakdown. In contrast, odds ratio estimates for Level-1 MLU were low (OR = 0.48), suggesting that individual change over time in MLU would not greatly impact probability of repair. Effect sizes, as calculated by odds ratio are provided in Table 4.

**Table 3.** Binomial multilevel models predicting probability of repair from language measures.

Model effects	Step 1: Empty means with random intercept		Step 2: Add random intercept for over-dispersion		Step 3: Add age		Step 4: Add autism and social skills	
	Est.	SE	Est.	SE	Est.	SE	Est.	SE
Model for the means								
Intercept	2.16**	0.20	4.07**	0.37	1.12	0.88	−0.56	1.69
Age in years					1.67**	0.51	1.53*	0.58
Quadratic age					−0.17**	0.06	−0.15*	0.06
MLU effects								
Level-2 between person							1.89^$^	1.02
Level-1 within person							−0.73^$^	0.42
NDW effects								
Level-2 between person							−0.02^$^	0.01
Level-1 within person							0.01	0.01
Model for the variance								
Random intercept variance	2.02**	0.47	1.18	1.09	1.42	1.10	1.17	1.06
Over-dispersion offset^a^			11.68**	2.27	10.54	2.09	10.44	2.11
−2 log likelihood	4582.40		1241.41		1229.63		1222.00	
AIC	4586.40		1247.41		1239.63		1240.00	
BIC	4590.38		1253.38		1249.57		1257.91	

SE: standard error; MLU: mean length of utterance; NDW: number of different words; AIC: Akaike information criterion; BIC: Bayesian information criterion.

^a^Over-dispersion offset is a proxy for residual variance in binomial models to account for additional variance relative to expected variance in a binomial distribution.

**p <* *0.01.

*p ≤* *0.05.

^$^*p *<* *0.10.

The addition of autism symptomology and social skills suggested that both were significant predictors of probability of repair, see Table 5. Level-2, or between-person effects of autism symptoms and VABS social skills were significant predictors, *F*(1,51) = 9.70, *p *=* *0.003, and *F*(1,51) = 8.20, *p *=* *0.006, respectively. Level-1, or within-person, effects were not significant *F*(1,127) = 0.55, *p *=* *0.46, and *F*(1,127) = 1.30, *p *=* *0.26. The effect sizes for Level-2 social predictors suggest that one unit increases in autism symptomology and social skills yield 1.36 and 1.15 greater likelihood of repair, respectively. Thus, children who had higher autism symptomology and higher social skills were more likely to repair. Effect sizes are reported in Table 4.

**Table 4.** Effect sizes for language and social predictors.

Predictor	Estimate (Odds ratio)	95% confidence interval
Age (years)	1.09	0.85–1.40
Level-2 MLU	6.64	0.85–51.50
Level-1 MLU	0.48	0.21–1.10
Level-2 NDW	0.98	0.96–1.00
Level-1 NDW	1.01	1.00–1.02
Level-2 CARS	1.36	1.11–1.65
Level-1 CARS	0.93	0.78–1.10
Level-2 VABS social	1.15	1.04–1.26
Level-1 VABS social	0.97	0.91–1.03

MLU: mean length of utterance; NDW: number of different words; VABS: Vineland Adaptive Behavior Scales; CARS: Childhood Autism Rating Scale.

Note: Odds ratio should be interpreted as the expected multiplicative increase in probability of repair for a one unit increase (10 unit increase for Level-2 NDW) in the predictor at age 2.5. Thus, the expected probability of repair is 6.64 times greater given an average (Level-2) MLU increase of 1 at age 2.5.

**Table 5.** Binomial multilevel models predicting probability of repair from social measures.

Model effects	Step 1: Empty means with random intercept		Step 2: Add random intercept for over-dispersion		Step 3: Add age		Step 4: Add autism and social skills	
	Est.	SE	Est.	SE	Est.	SE	Est.	SE
Model for the means								
Intercept	2.16**	0.20	4.07**	0.37	1.12	0.88	−0.56	1.69
Age in years					1.67**	0.51	1.53*	0.58
Quadratic age					−0.17**	0.06	−0.15*	0.06
CARS autism effects								
Level-2 between-person							0.30**	0.10
Level-1 within-person							−0.08	0.08
VABS social effects								
Level-2 between person							0.14**	0.05
Level-1 within person							−0.03	0.03
Model for the variance								
Random intercept variance	2.02**	0.47	1.18	1.09	1.42	1.10	0.36	0.92
Over-dispersion offset^a^			11.68**	2.27	10.54	2.09	11.02	2.28
−2 log likelihood	4582.40		1241.41		1229.63		1205.39	
AIC	4586.40		1247.41		1239.63		1223.39	
BIC	4590.38		1253.38		1249.57		1241.29	

SE: standard error; VABS: Vineland Adaptive Behavior Scales; CARS: Childhood Autism Rating Scale; AIC: Akaike information criterion; BIC: Bayesian information criterion.

^a^Over-dispersion offset is a proxy for residual variance in binomial models to account for additional variance relative to expected variance in a binomial distribution.

**p <* *0.01.

*p ≤* *0.05.

## Discussion

The development of repair in children with FXS was examined during naturalistic interactions between mother and child. Considering a mastery threshold of 90%, children with FXS reached mastery of repair by 3.5 years (42 months). Thus, development of repair was delayed relative to TD expectations, since children with TD demonstrate mastery by 14–21 months of age (Alexander, 1994; Gallagher, 1977). However, both the model predicted means and observed means suggest that once children acquire the ability to repair, they maintain this ability. Like children with TD, once children with FXS gain this pragmatic skill they are unlikely to lose it.

As children develop intentional communication, they begin to merge social skills with linguistic skills. They make use of speech, language, and social skills during social situations, and as such they need repair strategies to complement their maturing skills. We expected to see a predictive relationship between probability of repair and early expressive linguistic abilities. Our data suggest that emerging syntactic complexity, as measured by MLU in morphemes, and lexical diversity, as measured by NDW, were marginally predictive of the development of repair in children with FXS. We also predicted that social skills and autism symptomology would be significant predictors of repair. Indeed, social skills, measured by the VABS, and autism symptomology, measured by the CARS, were associated with probability of repair, suggesting that emerging social skills may also be an important predictor of repair.

To our knowledge, this is the first study of the development of repair in children with FXS and the only examination of repair in children with FXS in naturalistic settings. Although Roberts et al. (2002) described ability to repair based on a standardized assessment in young boys with FXS, they did not examine growth in this ability and did not include girls in their sample. Additionally, Roberts et al. (2002) did not consider the effect of autism symptomology or other predictors on ability to repair. Rather, they reported a relative weakness in repair in young boys with FXS, pointing toward potential impairments in reciprocity of social communication. While Barstein et al. (2018) examined differences in repair strategies among children with FXS-only, FXS and autism, autism, Down syndrome, and TD, their study only considered repair ability during structured assessment tasks, and again, did not consider developmental processes. They found that all groups demonstrated a robust ability to repair but recognized the limited generalizability of their structured task. Our study addressed these issues and found that the development of probability of repair was predicted by developing language and social skills in a naturalistic setting.

Repair has been suggested as an area of specific deficits in males with FXS with co-morbid autism (Barstein et al., 2018). Our multilevel regression models suggested that increased average autism symptomology was related to increased probability of repair. This relationship was unexpected. Unlike other studies, we did not differentiate our sample by sex or autism status. Indeed, when considering categorical co-morbid autism, there was no between-group difference. Repair and CARS scores were not significantly correlated, suggesting that the relationship identified in our prediction models may be due to Type I error. Perhaps this area of pragmatics is unaffected by the presence of autism symptoms in FXS, as previous studies have demonstrated that children and adolescents with FXS and autism have robust ability to repair (Barstein et al., 2018). Additionally, children with autism in the one-word stage repair 85% of the time, suggesting repair may not be reliant on expressive language skills in children with autism (Keen, 2005). Many of our participants with FXS and autism have limited verbal skills but persistent and robust nonverbal communication, which they may utilize during repair. Further research will be needed to disentangle the relationship between autism symptomology and repair in both FXS and idiopathic autism.

Opportunities to repair are contingent on partners’ signaling communication breakdowns. Importantly, in a naturalistic setting, mothers of children with FXS may work to ensure their child’s successful communication. This desire for child’s success may be borne out by mother’s only signaling communication breakdowns for which they believe their child can adequately repair. This may also explain the positive prediction of autism symptomology on repair. Mothers of children with FXS and high autism symptomology may be more cued into their child’s bids for communication and more sensitive their child’s communicative skills. As such, these mothers may adjust their own communication to accommodate their more severely impaired children and provide them with opportunities to repair. Comparison between naturalistic and assessment-derived settings may be additionally informative.

Our study may have been underpowered to detect significant predictive effects, as our language measures were marginally significant. Indeed, our small sample, in combination with a sizeable proportion of occasions with fewer than two repair opportunities and the limited variability in our outcome data, could have impacted our ability to detect significant effects. While we appropriately modeled probability of repair through binomial multilevel modeling, there was substantial skew towards 100% correct. We believe this skew was present due in part to the realization that once children acquire the ability to repair, they are unlikely to lose it. As such, there would be a skew in the data and limited variability. Multilevel models are designed to account for fixed and random effects, which require variation in the data. When this does not exist, the modeling may struggle to detect effects. Future studies wishing to examine growth in probability of repair should utilize larger datasets to ensure that sufficient variability exists. Thus, while our language findings were marginally significant, we believe that future studies that make use of larger datasets may be more informative. Finally, future studies of growth in repair should also consider adequacy of repair and complexity of repair. The current study considered attempts to repair but did not judge the adequacy or complexity of the repair. As such, it is unknown what proportion of repairs in this sample could be rated as successful, nor whether complexity of repairs changes with age in children with FXS.

The strengths of this study lie in the longitudinal nature of the data and the naturalistic setting in which data were collected. Participants in this ongoing longitudinal study have been visited 6 to 7 times from early toddlerhood into late childhood and now adolescence. The data in this study are from the toddlerhood through childhood visits, which enables us to detect patterns of growth in repair and language development. This study utilized pre-existing data that was collected during in-home data collection visits. As such, it provides us with an understanding of how repair are performed during naturalistic settings, rather than during standardized assessment procedures (Barstein et al., 2018). Although there are standardized assessments such as the Student Communication Repair Inventory & Practical Training (SCRIPT Inventory; Anderson, 2018), our data come from mother–child interactions during which communication breakdowns and opportunities to repair occur naturally. Naturalistic assessment of repair provides us with an understanding of how children with FXS are using repair strategies in daily activities with familiar communication partners. However, it prevents us from exploring how these children use repair strategies in novel situations or with new communication partners. It could be argued that the ability to repair in novel situations or with unknown communication partners is more important than when communicating with a familiar person such as a parent. Familiar communication partners or parents may more easily understand or interpret a child’s utterance than a stranger.

The ability to recognize a communication partner’s request for repair is an important pragmatic skill. The ability to adequately repair following this request is essential for conversational clarity and communicative competence. As such, clinicians working with children who may be at risk for delayed development of repair, such as children with FXS who demonstrate language delays or social skills impairments, must be able to address this skillset. We suggest that clinicians target this skill with all young children with FXS so that they can gain and master this skill as quickly as possible. One way to do this may be through protocols such as the SCRIPT Inventory (Anderson, 2018). This may be particularly relevant for clinicians working with children with FXS and high autism symptomology.

## References

[bibr1-2396941520909014] Abbeduto L. Brady N. Kover S. T. (2007). Language development and fragile X syndrome: Profiles, syndrome-specificity, and within-syndrome differences. Mental Retardation and Developmental Disabilities Research Reviews, 13, 36–46. 10.1002/mrdd.20142. 17326110 PMC7416600

[bibr2-2396941520909014] Abbeduto L. Murphy M. M. Kover S. T. Giles N. Karadottir S. Amman A. Bruno L. Kim J-S. Schroeder S. Anderson J. A. Nollin K. A. (2008). Signaling noncomprehension of language: A comparison of fragile X syndrome and down syndrome. American Journal on Mental Retardation, 113(3), 214–230. 10.1352/0895-8017(2008)113. 18407723 PMC5610960

[bibr3-2396941520909014] Alexander D. (1994). *The emergence of repair strategies in chronologically and developmentally young children*. [Unpublished doctoral dissertation]. Florida State University.

[bibr4-2396941520909014] Alexander D. Wetherby A. Prizant B. (1997). The emergence of repair strategies in infants and toddlers. Seminars in Speech and Language, 18(3), 197–212. 10.1055/s-2008-1064073. 9306516

[bibr5-2396941520909014] Anderson K. L. (2018). Repairing communication breakdowns in everyday situations: Student communication repair inventory & practical training (SCRIPT) (2nd ed.). Success for Children with Hearing Loss.

[bibr6-2396941520909014] ASHA. (2019). Social communication. Retrieved from https://www.asha.org/public/speech/development/social-communication/

[bibr7-2396941520909014] Barstein J. Martin G. E. Lee M. Losh M. (2018). A duck wearing boots?! Pragmatic language strategies for repairing communication breakdowns across genetically based neurodevelopmental disabilities. Journal of Speech Language Hearing Research, 61, 1440–1454. 10.1044/2018_JSLHR-L-17-0064. PMC6195092 29800075

[bibr8-2396941520909014] Brady N. McLean J. E. McLean L. K. Johnston S. (1995). Initiation and repair of intentional communication acts by adults with severe to profound cognitive disabilities. Journal of Speech and Hearing Research, 38, 1334–1348. 10.1044/jshr.3806.1334. 8747825

[bibr9-2396941520909014] Brady N. Steeples T. Fleming K. (2005). Effects of prelinguistic communication levels on initiation and repair of communication in children with disabilities. Journal of Speech, Language, and Hearing Research, 48(5), 1098–1113. 10.1044/1092-4388(2005/076). 16411798

[bibr10-2396941520909014] Brady N. Warren S. F. Fleming K. Keller J. Sterling A. (2014). The effect of sustained maternal responsivity on later vocabulary development in children with fragile X syndrome. Journal of Speech Language Hearing Research, 57(1), 212–226. 10.1044/1092-4388(2013/12-0341). PMC3864610 24023370

[bibr11-2396941520909014] Chen H. Cohen P. Chen S. (2010). How big is a big odds ratio? Interpreting the magnitudes of odds ratios in epidemiological studies. Communications in Statistics - Simulation and Computation, 39(4), 860–864. 10.1080/03610911003650383.

[bibr12-2396941520909014] Finestack L. Richmond E. Abbeduto L. (2009). Language development in individuals with fragile X syndrome. Topics in Language Disorders, 29(2), 133–148. 10.1097/TLD.0b013e3181a72016. 20396595 PMC2854517

[bibr13-2396941520909014] Gallagher T. M. (1977). Revision behaviors in the speech of normal children developing language. Journal of Speech and Hearing Research, 20, 303–318. 10.1044/jshr.2002.303. 895100

[bibr14-2396941520909014] Golinkoff R. M. (1986). ‘I beg your pardon?’: The preverbal negotiation of failed messages. Journal of Child Language, 13, 455–476. 10.1017/s0305000900006826. 3793809

[bibr15-2396941520909014] Grice H. P. (1975). Logic and conversation. In Cole P. Morgan J. L. (Eds.), Speech acts (pp. 41–58). Academic Press.

[bibr16-2396941520909014] Hoffman L. (2015). Longitudinal analysis: Modeling within-person fluctuation and change. Routledge Academic.

[bibr17-2396941520909014] Ialongo C. (2016). Understanding the effect size and its measures. Biochemia Medica, 26(2), 150–163. 10.11613/BM.2016.015. 27346958 PMC4910276

[bibr18-2396941520909014] Keen D. (2005). The use of non-verbal repair strategies by children with autism. Research in Developmental Disabilities, 26, 243–254. 10.1016/j.ridd.2004.07.002. 15668075

[bibr19-2396941520909014] Klusek J. Martin G. E. Losh M. (2014). A comparison of pragmatic language in boys with autism and fragile X syndrome. Journal of Speech Language Hearing Research, 57(5), 1692–1707. 10.1044/2014_JSLHR-L-13-0064. PMC4177023 24686468

[bibr20-2396941520909014] Lewis P. Abbeduto L. Murphy M. M. Richmond E. Giles N. Bruno L. Schroeder S. (2006). Cognitive, language and social-cognitive skills of individuals with fragile X syndrome with and without autism. Journal of Intellectual Disability Research, 50(7), 532–545. 10.1111/j.1365-2788.2006.00803.x. 16774638

[bibr21-2396941520909014] Martin G. E. Barstein J. Hornickel J. Matherly S. Durante G. Losh M. (2017). Signaling of noncomprehension in communication breakdowns in fragile X syndrome, Down syndrome, and autism spectrum disorder. Journal of Communication Disorders, 65, 22–34. 10.1016/j.jcomdis.2017.01.003. 28161297 PMC5340195

[bibr22-2396941520909014] Mazzocco M. M. Thompson L. Sudhalter V. Belser R. C. Lesniak-Karpiak K. Ross J. L. (2006). Language use in females with fragile X or Turner syndrome during brief initial social interactions. Developmental and Behavioral Pediatrics, 27(4), 319–328. 10.1097/00004703-200608000-00007. 16906008

[bibr23-2396941520909014] McDuffie A. Kover S. T. Abbeduto L. Lewis P. Brown T. (2012). Profiles of receptive and expressive language abilities in boys with comorbid fragile X syndrome and autism. American Journal on Intellectual and Developmental Disabilities, 117(1), 18–32. 10.1352/1944-7558-117.1.18. 22264110 PMC3265023

[bibr24-2396941520909014] Noldus Technology. (2002). The Observer, base package for Windows.

[bibr25-2396941520909014] Roberts J. E. Mirrett P. Anderson K. Burchinal M. Neebe E. (2002). Early communication, symbolic behavior, and social profiles of young males with fragile X syndrome. American Journal of Speech-Language Pathology, 11, 296–306. 10.1044/1058-0360(2002/034).

[bibr26-2396941520909014] Roberts J. E. Price J. Barnes E. Nelson L. Burchinal M. Hennon E. Moskowitz L. Edwards A. Malkin C. Anderson K. Misenheimer J. Hooper S. R. (2007). Receptive vocabulary, expressive vocabulary, and speech production of boys with fragile X syndrome in comparison to boys with Down syndrome. American Journal on Mental Retardation, 112(3), 177–193. 10.1352/0895-8017(2007)112 17542655

[bibr27-2396941520909014] SAS Institute Inc. (2013). *SAS 9.4*.

[bibr28-2396941520909014] Schopler E. Reichler R. J. Renner B. R. (1988). The childhood autism rating scale. Western Psychological Services.

[bibr29-2396941520909014] Sparrow S. S. Balla D. A. Cicchetti D. (1984). Vineland Adaptive Behavior Scales. American Guidance Service.

[bibr30-2396941520909014] Sparrow S. S. Cicchetti D. Balla D. A. (2005). Vineland-II: Vineland Adaptive Behavior Scales. (2nd ed.). Pearson.

[bibr31-2396941520909014] Sterling A. Abbeduto L. (2012). Language development in school-age females with fragile X syndrome. Journal of Intellectual Disability Research, 56(10), 974–983. 10.1111/j.1365-2788.2012.01578.x. 22676254 PMC3627376

[bibr32-2396941520909014] Warren S. F. Brady N. Sterling A. Fleming K. Marquis J. (2010). Maternal responsivity predicts language development in young children with fragile X syndrome. American Journal of Intellectual and Developmental Disabilities, 115(1), 54–75. 10.1352/1944-7558-115.1.54. PMC3045825 20025359

[bibr33-2396941520909014] Wetherby A. Prizant B. (2003). Communication and Symbolic Behaviors Scales manual. Applied Symbolix.

[bibr34-2396941520909014] Yoder P. J. Watson L. R. Lambert W. (2015). Value-added predictors of expressive and receptive language growth in initially nonverbal preschoolers with autism spectrum disorders. Journal of Autism and Developmental Disorders, 45(5), 1254–1270. 10.1007/s10803-014-2286-4. 25344152 PMC4495651

